# Association between chronic diseases and severe periodontal disease progression: A retrospective cohort study in a city of Japan

**DOI:** 10.1002/jgf2.734

**Published:** 2024-10-04

**Authors:** Sayaka Nin, Yu Sun, Takami Maeno, Chihiro Nishiura, Kento Taira, Kenji Fujimoto, Jun Hamano, Sachiko Ozone, Tetsuhiro Maeno

**Affiliations:** ^1^ Graduate School of Comprehensive Human Sciences University of Tsukuba Ibaraki Japan; ^2^ Department of Primary Care and Medical Education, Institute of Medicine University of Tsukuba Ibaraki Japan; ^3^ Wako Human Resources Section RIKEN Wako Japan; ^4^ Health Services Research and Development Center University of Tsukuba Ibaraki Japan; ^5^ Occupational Health Data Science Center University of Occupational and Environmental Health Kitakyushu Japan; ^6^ Institute of Medicine University of Tsukuba Ibaraki Japan

**Keywords:** chronic diseases, diabetes mellitus, periodontal diseases, population‐based survey, primary health care

## Abstract

**Background:**

Chronic periodontal disease primarily causes tooth loss and oral frailty and is linked to chronic conditions such as diabetes mellitus. However, its progression and broader studies on chronic diseases have not been well explored. This study aimed to investigate this association using claims data.

**Methods:**

This retrospective cohort study used linked medical, dental, and pharmacy claims data from a local municipality in Japan. The study included participants aged 40–70 years who had received medical care between April 2017 and March 2018. Exposures included age, sex, and common chronic diseases previously reported to be associated with periodontal diseases (21 diseases). We defined the outcome, “progression of severe periodontitis” as the worsening of periodontal disease to a severe stage requiring surgery or tooth extraction, determined by the presence of a periodontal surgery code or a deeper probing pocket depth (≥6 mm) code along with the tooth extraction procedure code. The participants were followed up until March 2022, and multivariate analysis was conducted using Cox proportional hazard models.

**Results:**

Among 28,846 participants, 1035 (3.6%) progressed to severe periodontal disease. In the multivariate analysis, only diabetes mellitus was significantly associated with severe periodontal disease, with a hazard ratio of 1.26 (95% confidence interval, 1.08–1.53) among all chronic diseases.

**Conclusion:**

Patients with diabetes mellitus had a high risk of severe periodontal disease progression, suggesting that proactive dental visits should be recommended to prevent severe periodontal disease.

## Graphical abstract

This manuscript is a study demonstrating the association between the progression of severe periodontal diseases and chronic diseases commonly addressed in primary care. We used claims data and defined the outcome as the “progression of severe periodontitis” and followed patients for 4 years. As a result, patients with diabetes mellitus had a higher risk of severe periodontal disease progression.

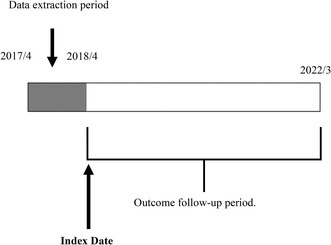

## 1 INTRODUCTION

Chronic periodontal disease involves complex interactions between oral bacteria and the overall health of patients. Severe periodontal disease, resulting in the dissolution of the alveolar bone and loss of tooth support, which leads to eventual tooth loss, is a significant concern. Tooth loss contributes to oral frailty (including chewing problems), which in turn affects overall health^1^ and quality of life. Since the dissolved alveolar bone does not regenerate naturally, it is important to prevent severe periodontal disease.

Successful management of periodontal disease may improve the quality of life of patients.^2^, ^3^ In Japan, 49% of adults have periodontal disease,^4^ with the percentage increasing with age; only 55% have been reported to have visited a dentist in the past year. Consequently, identifying individuals prone to periodontal disease and promoting early dental interventions are crucial.

Previous studies have demonstrated associations between periodontal disease and chronic diseases, including diabetes mellitus, hypertension,^5^ dyslipidemia,^6^ chronic kidney disease (CKD),^7^ chronic obstructive pulmonary disease (COPD),^8^ cardiovascular diseases (CVDs),^9^, ^10^, ^11^ rheumatoid arthritis (RA),^12^ inflammatory bowel disease (IBD),^13^ osteoporosis,^14^ obesity,^15^ and hepatitis/cirrhosis.^16^ In this context, diabetes mellitus has garnered significant attention owing to its correlation with periodontal diseases.^17^, ^18^

However, previous studies present two problems. First, considering that periodontal disease affects half the population and that some patients with periodontal disease are less likely to progress to severe periodontitis,^19^ it is crucial to focus on the progression of severe periodontal disease rather than mild cases. Second, although preventing the progression of severe periodontal disease is crucial, cohort studies on causal relationships are limited. To the best of our knowledge, no cohort study has been published that includes a wide range of common chronic diseases as potential risk factors for the progression of severe periodontal disease. Therefore, this study aimed to investigate the association between the progression of severe periodontal disease and common chronic diseases using medical, dental, and pharmacy claims data obtained from a local municipality in Japan.

## 2 METHODS

### 2.1 Data source

We obtained linked medical, dental, and pharmacy claims data from individuals with National Health Insurance for the self‐employed and retirees aged <75 years in Mito City (population approximately 270,000, prefectural capital, around 63,000 people were enrolled in National Health Insurance^20^) in Ibaraki Prefecture, covering the period from April 2017 to March 2022. Medical and dental claims records included monthly information on diagnoses and medical procedures. Recorded diagnoses were based on the original Japanese disease codes linked to the International Classification of Diseases, Tenth Revision (ICD‐10) codes, and the Japanese Standard Disease Code Master. Medical procedures were coded using the original Japanese codes. Pharmacy claims data included prescribed medical drugs based on the Ministry of Health, Labor and Welfare (MHLW) drug codes.^21^ The MHLW drug codes are those managed by the MHLW for the drug price standard and feature 12‐digit alphanumeric drug codes.

The municipal government links medical, dental, and pharmacy claims data using personally identifiable information. Anonymized ID numbers were assigned to individuals in the medical, dental, and pharmacy claims datasets. The data include all records of individuals enrolled in the National Health Insurance.

### 2.2 Research design and population

**FIGURE 1:**
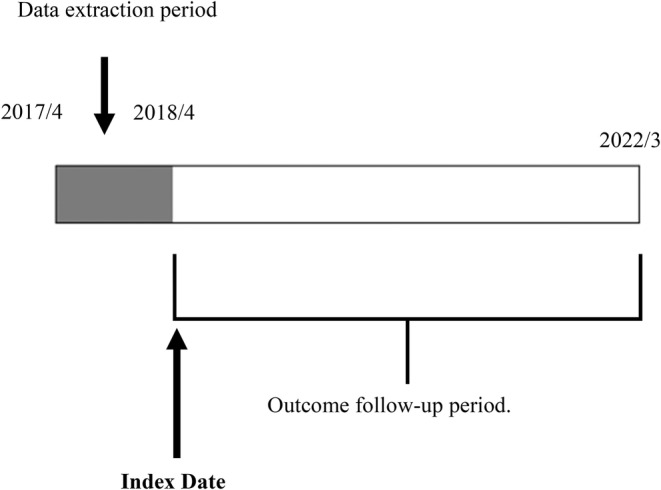
Diagram of data extraction period and index date.

This retrospective cohort study was conducted using a previously mentioned database. The target population included all individuals in Mito City who were enrolled in the National Health Insurance program. The inclusion criteria were as follows: participants aged 40–70 years in 2017 and those who attended medical care between April 1, 2017, and March 31, 2018 (the data extraction period). Patients who developed severe periodontal disease during the data extraction period were excluded (Figure 1). We set an index date of April 1, 2018, and the primary outcome was monitored for 4 years until March 31, 2022 (Figure 1).

We chose the age range because “Survey of Dental Disease Fiscal Year 2022”^22^ by MHLW indicates that the number of patients with severe periodontitis increases from age 40 and above, and there are modifications in the Japanese health insurance system after patients turn 75 years old, which makes it difficult to track them using claims data. Therefore, we limited the initial age range to 40–70 years.

### 2.3 Exposure

Exposure factors were age (40–49, 50–59, and 60–70), sex, common chronic diseases,^23^ and chronic conditions previously reported to be associated with periodontal diseases.^7^, ^8^, ^9^, ^10^, ^11^, ^12^, ^13^, ^14^, ^15^, ^16^ These included diabetes mellitus, hypertension, dyslipidemia, chronic heart failure, CKD, COPD, asthma, CVDs, gastric disorders (chronic gastritis and gastroesophageal reflux disease), IBD, hepatitis/cirrhosis, cancer, obesity, RA, arthralgia, osteoporosis, chronic musculoskeletal conditions, dementia, anxiety/depression, thyroid disease, and urinary tract diseases. To improve the accuracy of identifying patients with dyslipidemia, hypertension, and diabetes mellitus, which are highly prevalent in Japan,^24^ we defined each disease based on the combination of prescription and ICD‐10 codes in the same month, following previous study algorithms^25^ (sensitivity/specificity of this algorithm: hypertension, 74.5%/98.2%; diabetes mellitus, 78.6%/99.6%; and dyslipidemia, 34.5%/97.2%). Other diseases were defined using the ICD‐10 codes. The ICD‐10 and drug codes are listed in Appendices 1 and 2, respectively. These exposure factors were extracted during the data extraction period (April 1, 2017 to March 31, 2018). If these criteria were met at least once during this period, the participant was defined as having a chronic disease.

### 2.4 Outcome

The initial extraction date for the progression of severe periodontal disease was defined as the date of outcome occurrence. The incidence of severe periodontal disease was tracked over 4 years, spanning from April 1, 2018 to March 31, 2022, with the index date set at April 1, 2018.

Perigingival inflammation results in the widening of the gap between the gingiva and tooth root and course pocket. The severity of periodontal disease is classified based on pocket depth.^26^ In Japan, the severity of periodontal disease is classified based on probing pocket depth, with mild as P1 (pocket <4 mm), moderate as P2 (pocket <6 mm), and severe as P3 (probing pocket depth ≥6 mm). In Japanese dental claims data, P1 and P2 are collectively labeled as “P,” while severe periodontal disease requiring tooth extraction is labeled as “P3.” The occurrence of “P3” along with a tooth extraction procedure indicates severe periodontal disease. Furthermore, the periodontal surgical code indicates severe periodontal disease. For these reasons, the progression of severe periodontal disease in this study was defined as either periodontal surgery (medical procedure code: J063) or P3 disease (Japanese Standard Disease Code Master: 8843616) combined with an extraction procedure code (medical procedure code: J000). This definition was determined by the authors, who included dentists with experience in claims data research.

### 2.5 Statistical analysis

The basic characteristics are expressed as percentages. Hazard ratios (HRs) and 95% confidence intervals (CIs) were calculated using univariate and multivariate Cox regression analyses to assess which chronic diseases had a greater impact on the risk of developing severe periodontal disease. The variables included age, sex, diabetes mellitus, hypertension, dyslipidemia, chronic heart failure, CKD, COPD, asthma, CVDs, gastric disorders, IBD, hepatitis/cirrhosis, cancer, obesity, RA, arthralgia, osteoporosis, chronic musculoskeletal conditions, dementia, anxiety/depression, thyroid disease, and urinary tract disease. All variables were included in multivariable analysis. In addition, we conducted a sensitivity analysis to examine the influence of a history of regular dental examinations. We extracted a group without dental examinations during the data extraction period, and the Cox regression analysis was performed in the same manner.

All statistical analyses were performed using STATA version 17.0 (Stata Corp., College Station, TX, USA). Statistical significance was set at *p* < 0.05.

### 2.6 Ethical approval

This study was approved by the Ethics Committee of the University of Tsukuba (approval number: 1767). The requirement for consent from individual participants was waived because of the anonymity of the data.

## 3 RESULTS

### 3.1 Study population and baseline characteristics

Among the initially included 29,002 patients, 156 with severe periodontal disease during the data extraction period were excluded, resulting in a total of 28,846 patients being included in the analysis. Table 1 shows the number and incidence of primary outcomes categorized according to age group, sex, and chronic disease. Chronic musculoskeletal conditions have emerged as the most prevalent chronic conditions, followed by hypertension, gastric disorders, and dyslipidemia. During the 4‐year follow‐up period, 1035 (3.6%) patients developed severe periodontal disease.

**TABLE 1:** Participants' characteristics.

	All patients (%)	With severe periodontal disease (%)	Without severe periodontal disease (%)
	*N* = 28,846	*N* = 1035	*N* = 27,811
	*n* (%)	*n* (%)	*n* (%)
Age (years)			
40–49	4955 (17.2)	92 (8.9)	4863 (17.5)
50–59	5240 (18.2)	141 (13.6)	5099 (18.3)
60–70	18,651 (64.7)	802 (77.5)	17,849 (64.2)
Sex			
Female	15,714 (54.5)	525 (50.7)	15,189 (54.6)
Chronic disease			
Diabetes mellitus	3790 (13.1)	180 (17.4)	3610 (13.0)
Hypertension	8236 (28.6)	332 (32.1)	7904 (28.4)
Dyslipidemia	7159 (24.8)	285 (27.5)	6874 (24.7)
Chronic heart failure	1913 (6.6)	82 (7.9)	1831 (6.6)
CKD	635 (2.2)	25 (2.4)	610 (2.2)
COPD	429 (1.5)	19 (1.8)	410 (1.5)
Asthma	2822 (9.8)	92 (8.9)	2730 (9.8)
CVDs	4782 (16.6)	210 (20.3)	4572 (16.4)
Gastric disorders	7847 (27.2)	298 (28.8)	7549 (27.1)
IBD	146 (0.5)	5 (0.5)	141 (0.5)
Hepatitis/cirrhosis	4194 (14.5)	170 (16.4)	4024 (14.5)
Cancer	1925 (6.7)	79 (7.6)	1846 (6.6)
Obesity	225 (0.8)	6 (0.6)	219 (0.8)
Rheumatoid arthritis	667 (2.3)	26 (2.5)	641 (2.3)
Arthralgia	2672 (9.3)	107 (10.3)	2565 (9.2)
Osteoporosis	2323 (8.1)	88 (8.5)	2235 (8.0)
Chronic musculoskeletal conditions	9156 (31.7)	346 (33.4)	8810 (31.7)
Dementia	178 (0.6)	4 (0.4)	174 (0.6)
Anxiety/depression	1298 (4.5)	46 (4.4)	1252 (4.5)
Thyroid disease	1289 (4.5)	53 (5.1)	1236 (4.4)
Urinary tract disease	3402 (11.8)	141 (13.6)	3261 (11.7)

Abbreviations: CKD, chronic kidney disease; COPD, chronic obstructive pulmonary disease; CVDs, cardiovascular disease; IBD, inflammatory bowel disease.

### 3.2 Univariate analysis

The univariate analysis revealed significant associations between the progression of severe periodontal disease and various factors, including old age, male sex, diabetes mellitus, hypertension, dyslipidemia, chronic heart failure, CVDs, gastric disorders, hepatitis/cirrhosis, cancer, arthralgia, chronic musculoskeletal conditions, and urinary tract disease (Table 2).

**TABLE 2:** Univariate and multivariate analysis for severe periodontal disease.

	Univariate		Multivariate^a^	
	HRs (95% CIs)	*p*‐Value	HRs (95% CIs)	*p*‐Value
Age (years)				
40–49 (reference)	1.00	–	1.00	–
50–59	1.46 (1.12–1.89)	0.005	1.43 (1.10–1.87)	0.008
60–70	2.34 (1.89–2.91)	<0.001	2.29 (1.83–2.86)	<0.001
Sex (reference = female)	1.17 (1.03–1.32)	0.014	1.14 (1.00–1.30)	0.047
Chronic diseases^b^				
Diabetes mellitus	1.41 (1.19–1.65)	<0.001	1.26 (1.08–1.53)	0.008
Hypertension	1.19 (1.04–1.35)	0.101	0.97 (0.84–1.50)	0.643
Dyslipidemia	1.15 (1.01–1.32)	0.039	0.96 (0.83–1.11)	0.567
Chronic heart failure	1.21 (0.97–1.53)	0.086	1.04 (0.81–1.33)	0.754
CKD	1.11 (0.74–1.66)	0.597	0.93 (0.61–1.41)	0.723
COPD	1.24 (0.79–1.96)	0.340	1.10 (0.69–1.75)	0.686
Asthma	0.90 (0.72–1.11)	0.325	0.88 (0.70–1.09)	0.247
CVDs	1.29 (1.11–1.50)	0.001	1.11 (0.94–1.32)	0.219
Gastric disorders	1.08 0.95–1.24)	0.237	0.95 (0.82–1.10)	0.503
IBD	0.95 (0.40–2.30)	0.913	0.98 (0.42–2.43)	0.970
Hepatitis/cirrhosis	1.16 (0.98–1.37)	0.075	1.06 (0.89–1.26)	0.494
Cancer	1.16 (0.92–1.46)	0.214	1.02 (0.81–1.29)	0.861
Obesity	0.74 (0.33–1.64)	0.456	0.78 (0.35–1.75)	0.547
Rheumatoid arthritis	1.09 (0.74–1.61)	0.655	1.06 (0.71–1.59)	0.755
Arthralgia	1.13 (0.93–1.38)	0.228	1.06 (0.85–1.30)	0.619
Osteoporosis	1.6 (0.86–1.32)	0.574	0.95 (0.75–1.21)	0.690
Chronic musculoskeletal conditions	1.08 (0.95–1.23)	0.223	1.02 (0.88–1.17)	0.801
Dementia	0.62 (0.23–1.66)	0.346	0.52 (0.19–1.38)	0.187
Anxiety/depression	0.99 (0.73–1.33)	0.933	1.04 (0.77–1.40)	0.797
Thyroid disease	1.16 (0.88–1.53)	0.294	1.12 (0.85–1.49)	0.417
Urinary tract disease	1.18 (0.99–1.42)	0.061	1.04 (0.86–1.25)	0.672

Abbreviations: CIs, confidence intervals; CKD, chronic kidney disease; COPD, chronic obstructive pulmonary disease; CVDs, cardiovascular disease; HRs, Hazard ratios; IBD, inflammatory bowel disease.

^a^ All variables were included in multivariable analysis.

^b^ Chronic diseases were referenced as “no disease”.

### 3.3 Multivariate analysis

In the multivariate analysis encompassing all variables, individuals aged 50–59 years (HR [95% CI], 1.43 [1.10–1.87]) and 60–70 years (2.29 [1.83–2.86]) had a significantly higher incidence of severe periodontal disease progression than those aged 40–49 years. Moreover, males (1.14 [1.00–1.30]) and patients with diabetes mellitus (1.26 [1.08–1.53]) had a significantly high incidence of severe periodontal disease progression (Table 2).

### 3.4 Sensitivity analysis

A total of 18,873 patients had no history of dental examinations during the data extraction period. This accounted for 65% of the total study population. Of these, 478 (2.5%) individuals developed severe periodontal disease in the following 4 years. The multivariate Cox regression analysis showed a significant association of severe periodontal disease progression with diabetes mellitus (HR, 1.60 [95% CI, 1.27–2.02]), in addition to old age, mostly consistent with the main results (Appendices 3 and 4).

## 4 DISCUSSION

To the best of our knowledge, this is the first study to investigate the association between chronic disease and severe periodontal disease progression using linked medical, dental, and pharmacy claims data obtained from a local municipality in Japan. The most important finding of our study was that patients with diabetes mellitus requiring medication had a high risk of severe periodontal disease progression (HR, 1.26 [95% CI, 1.08–1.53]).

Our finding that patients with diabetes mellitus, particularly those requiring treatment, are at a high risk of severe periodontal disease progression is consistent with the results of a previous study.^18^ A possible mechanism^27^ is that diabetes mellitus influences periodontitis by triggering hyperinflammatory responses and impairing bone repair, leading to accelerated periodontal destruction.

In contrast, no chronic condition other than diabetes mellitus showed a significant association in our study. Previous studies have reported an association between periodontal diseases and various chronic conditions. For example, a study^14^ using claims data with a multivariate analysis identified associations between hypertension, CVDs, RA, and periodontal disease. Another study^28^ found an association between diabetes mellitus, hypertension, and the progression of periodontal disease. However, the study had several limitations, such as not including a sufficient range of chronic diseases, aggregating all periodontal conditions regardless of severity, and relying on self‐reported data for chronic diseases, which may have reduced validity. Therefore, our focus on severe periodontal disease and comprehensive adjustment for chronic diseases may have allowed for highly accurate identification of the most important factors affecting periodontal progression.

Our results imply that the risk of severe periodontal disease progression is not only associated with diabetes mellitus but also with old age and male sex. Several studies have reported that old age^26^ and male sex^29^ are associated with periodontal disease, which is consistent with our findings. Two potential explanations can be hypothesized: (1) old age^26^ and male sex^29^ are inherent risk factors and (2) factors such as smoking or metabolic syndrome,^29^ already acknowledged as risk factors for periodontitis, may act as confounding factors.

In the sensitivity analysis, the group that did not undergo dental examinations during the data collection period showed similar results. Ideally, recommendations for dental visits should target individuals who do not usually visit a dentist. However, considering that 65% of the participants in our study had no regular dental examinations and both the main and sensitivity analyses yielded consistent results, it is reasonable to recommend dental visits to individuals with risk factors without necessarily confirming their history of dental visits in a busy clinical setting.

Our study had two strengths. First, we included 21 chronic diseases that were objectively diagnosed, including those commonly encountered. This enabled us to comprehensively explore its relationship with the progression of severe periodontal diseases. Second, we conducted a cohort study with follow‐up for 4 years. Compared with cross‐sectional studies, this approach strengthens our understanding of the causal relationship regarding the progression of periodontal diseases.

However, this study had some limitations. First, certain chronic conditions were extracted solely based on ICD‐10 disease codes because of a lack of validation studies using a combination of drug codes, potentially leading to low sensitivity and specificity. Second, because we did not have information on insurance changes in our data, we could not track discontinuations. Additionally, owing to the inability to verify “death” in the database, we did not analyze the competing risk of death. However, because all participants were below the age of 70, we concluded that the impact of death was low. Third, we were unable to include patients with periodontal disease who did not visit a dentist; therefore, some cases may have been overlooked. In addition, although we excluded individuals who underwent severe periodontitis treatment within the past year (data extraction period), their prior conditions remain unknown. Older individuals may have had an increased likelihood of receiving treatment for severe periodontitis in the past, or they may have fewer remaining teeth, which could be a confounding factor. Fourth, although factors such as smoking, socioeconomic status, or control of chronic diseases may have influenced the results, they could not be considered because of insufficient information in the claims data. The municipality conducts regular health checkups, where smoking status and blood test results are recorded. Therefore, a precise study would be possible if this information were available. Fifth, this study used data from a single municipality, which limits its generalizability. Further validation using a broader range of data, such as data from all prefectures, is necessary.

In conclusion, considering the wide range of chronic diseases in this study, we found that patients with diabetes mellitus requiring medication had a high risk of severe periodontal disease progression. Additionally, old age and male sex were identified as risk factors. Considering that 65% of individuals had a medical history but no dental visits within the past year, there is a significant need to promote dental checkups by physicians. Our study findings suggest that physicians who manage chronic diseases should encourage dental visits, particularly for patients with diabetes mellitus and male and older patients, to prevent the progression of severe periodontal disease.

## CONFLICT OF INTEREST STATEMENT

The other authors have stated explicitly that here are no conflicts of interest in connection with this article.

## ETHICS STATEMENT

Ethics approval statement: This study was approved by the Ethics Committee of the University of Tsukuba (approval number: 1767).

Patient consent statement: None.

Clinical trial registration: None.

## APPENDIX 1 International Classification of Diseases, Tenth Revision codes of all chronic diseases used as exposure factors

### 1.1

**Table:**

Chronic diseases	ICD‐10 codes
Diabetes mellitus	E10–14
Hypertension	I10–I15
Dyslipidemia	E78
Chronic heart failure	I05–I09, I34–I39, I42 I43, I50
CKD	N18, N19
COPD	J43, J44
Asthma	J41, J42, J45, J46
CVDs	I20–I25, I70–I79, I60–I63
Gastric disorders	K21, K25.7, K29.5
IBD	K50–K52
Hepatitis/cirrhosis	K70–K77
Cancer	C00–C97
Obesity	E66
Rheumatoid arthritis	M05, M06
Arthralgia	M13.0, M13.9, M15–M19
Osteoporosis	M81
Chronic musculoskeletal conditions	M40–M54, M60–M63, M65–M68, M70–M79
Dementia	F00–F03, G30
Anxiety/depression	F40, F41, F33
Thyroid disease	E00–E07
Urinary tract disease	N03, N11, N20–N23, N25–N29, N30–N39, N40–N51

Abbreviations: CKD, chronic kidney disease; COPD, chronic obstructive pulmonary disease.

## APPENDIX 2 Drug list for diabetes mellitus, hypertension, and dyslipidemia

### 2.1

**Table:**

The type of medicine for diabetes
Acarbose
Acetohexamide
Alogliptin benzoate
Alogliptin benzoate/Pioglitazone hydrochloride
Alogliptin benzoate/Metformin hydrochloride
Anagliptin
Anagliptin/Metformin hydrochloride
Buformin hydrochloride
Buformin hydrochloride
Canagliflozin hydrate
Chlorpropamide
Dapagliflozin propylene glycolate hydrate
Empagliflozin
Empagliflozin/Linagliptin
Glibenclamide
Glimepiride
Glyclopyramide
Imeglimin hydrochloride
Ipragliflozin L‐proline
Linagliptin
Luseogliflozin hydrate
Metformin hydrochloride
Miglitol
Mitiglinide calcium hydrate
Mitiglinide calcium hydrate/Voglibose
Nateglinide
Omarigliptin
Pioglitazone hydrochloride
Pioglitazone hydrochloride/Metformin hydrochloride
Pioglitazone hydrochloride/Glimepiride
Repaglinide
Saxagliptin hydrate
Semaglutide
Sitagliptin phosphate hydrate
Sitagliptin phosphate hydrate/Ipragliflozin L‐proline
Teneligliptin hydrobromide hydrate
Teneligliptin hydrobromide hydrate/Canagliflozin hydrate
Tofogliflozin hydrate
Tolbutamide
Trelagliptin succinate
Vildagliptin
Vildagliptin/Metformin hydrochloride
Voglibose
Insulin (human)
Insulin lispro
Insulin aspart
Insulin glargine
Insulin detemir
Insulin glulisine
Insulin degludec
Insulin glargine
Insulin lispro
Insulin degludec/Insulin aspart
Liraglutide
Exenatide
Lixisenatide
Dulaglutide
Semaglutide
Teduglutide
Tirzepatide
Insulin degludec/Liraglutide
Insulin glargine/Lixisenatide
The type of medicine for hypertension
Alacepril
Aliskiren fumarate
Azelnidipine
Azilsartan
Azilsartan/Amlodipine besilate
Barnidipine hydrochloride
Benazepril hydrochloride
Benidipine hydrochloride
Candesartan cilexetil
Candesartan cilexetil/Amlodipine besilate
Candesartan cilexetil/Hydrochlorothiazide
Captopril
Cilazapril hydrate
Cilnidipine
Delapril hydrochloride
Diltiazem hydrochloride
Efonidipine hydrochloride ethanolate
Enalapril maleate
Eplerenone
Felodipine
Imidapril hydrochloride
Irbesartan
Irbesartan/Amlodipine besilate
Lisinopril hydrate
Losartan potassium
Losartan potassium/Hydrochlorothiazide
Manidipine hydrochloride
Nicardipine hydrochloride
Nifedipine
Nilvadipine
Nisoldipine
Nitrendipine
Olmesartan medoxomil
Olmesartan medoxomil/Azelnidipine
Perindopril erbumine
Quinapril hydrochloride
Reserpine/Hydralazine hydrochloride/Hydrochlorothiazide
Sacubitril valsartan sodium hydrate
Telmisartan
Telmisartan/Amlodipine besilate
Telmisartan/Amlodipine besilate/Hydrochlorothiazide
Telmisartan/Hydrochlorothiazide
Temocapril hydrochloride
Trandolapril
Urapidil
Valsartan
Valsartan/Amlodipine besilate
Valsartan/Cilnidipine
Valsartan/Hydrochlorothiazide
Verapamil hydrochloride
The type of medicine for dyslipidemia
Atorvastatin calcium hydrate
Bezafibrate
Clofibrate
Colestimide
Colestyramine
Dextran sulfate sodium sulfur
Elastase
Ethyl icosapentate
Evolocumab (genetical recombination)
Ezetimibe
Ezetimibe/Atorvastatin calcium hydrate
Ezetimibe/Rosuvastatin calcium
Fenofibrate
Fluvastatin sodium
Gamma oryzanol
Lomitapide mesilate
Niceritrol
Nicomol
Omega‐3‐Acid ethyl esters
Pemafibrate
Pitavastatin calcium hydrate
Pitavastatin calcium hydrate/Ezetimibe
Polyenephosphatidylcholine
Pravastatin sodium
Probucol
Rosuvastatin calcium
Simvastatin

## APPENDIX 3 Characteristics of participants without a history of dental examination during the data extraction period

### 3.1

**Table:**

	All patients (%)	With severe periodontal disease (%)	Without severe periodontal disease (%)
	*N* = 18,873	*N* = 478	*N* = 18,395
	*n* (%)	*n* (%)	*n* (%)
Age (years)			
40–49	3417 (18.1)	47 (9.8)	3370 (18.3)
50–59	3540 (18.8)	76 (15.9)	3640 (19.8)
60–70	11816 (62.6)	355 (74.3)	11461 (62.3)
Sex			
Female	9800 (51.9)	224 (46.9)	9576 (52.1)
Chronic disease			
Diabetes mellitus	2653 (14.1)	108 (22.6)	2545 (13.8)
Hypertension	5623 (29.8)	177 (37.0)	5446 (29.6)
Dyslipidemia	4642 (24.6)	138 (28.9)	4504 (24.5)
Chronic heart failure	1311 (6.9)	45 (9.4)	1266 (6.9)
CKD	446 (2.4)	10 (2.1)	436 (2.4)
COPD	308 (1.6)	12 (2.5)	296 (1.6)
Asthma	1823 (9.7)	41 (8.6)	1782 (9.7)
CVDs	3185 (16.9)	99 (20.7)	3086 (16.8)
Gastric disorders	4919 (26.1)	133 (27.8)	4786 (26.0)
IBD	92 (0.5)	2 (0.4)	90 (0.5)
Hepatitis/cirrhosis	2730 (14.5)	84 (17.6)	2646 (14.4)
Cancer	1206 (6.4)	44 (9.2)	1162 (6.3)
Obesity	151 (0.8)	3 (0.6)	148 (0.8)
Rheumatoid arthritis	425 (2.3)	15 (3.1)	410 (2.2)
Arthralgia	1620 (8.6)	51 (10.7)	1569 (8.5)
Osteoporosis	1377 (7.3)	36 (7.5)	1341 (7.3)
Chronic musculoskeletal conditions	5834 (30.9)	163 (34.1)	5671 (30.8)
Dementia	132 (0.7)	3 (0.6)	129 (0.7)
Anxiety/depression	805 (4.3)	22 (4.6)	783 (4.3)
Thyroid disease	803 (4.3)	24 (5.0)	779 (4.2)
Urinary tract disease	2113 (11.2)	71 (14.9)	2062 (11.2)

Abbreviations: CKD, chronic kidney disease; COPD, chronic obstructive pulmonary disease; CVDs, cardiovascular disease; IBD, inflammatory bowel disease.

## APPENDIX 4 Multivariate analysis for severe periodontal disease in individuals without a history of dental examination during the data extraction period

### 4.1

**Table:**

	Hazard ratios	95% confidence intervals		*p*‐Value
Age (years)				
40–49 (reference)	1.00	–	–	–
50–59	1.42	0.99	2.06	0.056
60–70	1.93	1.41	2.65	<0.001
Sex (reference = female)	1.15	0.96	1.41	0.113
Chronic diseases^a^				
Diabetes mellitus	1.60	1.27	2.02	<0.001
Hypertension	1.11	0.90	1.36	0.319
Dyslipidemia	0.97	0.79	1.21	0.843
Chronic heart failure	1.20	0.86	1.67	0.292
CKD	0.62	0.32	1.19	0.148
COPD	1.34	0.74	2.43	0.328
Asthma	0.83	0.59	1.15	0.263
CVDs	1.01	0.79	1.30	0.917
Gastric disorders	0.90	0.72	1.12	0.341
IBD	0.86	0.21	3.46	0.831
Hepatitis/cirrhosis	1.07	0.84	1.36	0.600
Cancer	1.33	0.97	1.83	0.076
Obesity	0.74	0.24	2.30	0.600
Rheumatoid arthritis	1.40	0.82	2.37	0.219
Arthralgia	1.19	0.87	1.62	0.273
Osteoporosis	0.91	0.63	1.31	0.613
Chronic musculoskeletal conditions	1.05	0.85	1.28	0.669
Dementia	0.75	0.24	2.33	0.613
Anxiety/depression	1.15	0.74	1.77	0.532
Thyroid disease	1.12	0.73	1.69	0.607
Urinary tract disease	1.16	0.90	1.51	0.257

Abbreviations: CKD, chronic kidney disease; COPD, chronic obstructive pulmonary disease; CVDs: cardiovascular diseases.

^a^Chronic diseases referenced as “no disease”.
